# An extremely rich repertoire of bursting patterns during the development of cortical cultures

**DOI:** 10.1186/1471-2202-7-11

**Published:** 2006-02-07

**Authors:** Daniel A Wagenaar, Jerome Pine, Steve M Potter

**Affiliations:** 1 Department of Physics, California Institute of Technology, Caltech 103-33, Pasadena, CA 91125, USA; 2 Department of Physics, California Institute of Technology, Caltech 256-48, Pasadena, CA 91125, USA; 3 Coulter Department of Biomedical Engineering, Georgia Institute of Technology and Emory University, 313 Ferst Drive, Atlanta, GA 30332-0535, USA; 4 Present address: Division of Biological Sciences, University of California at San Diego, 9500 Gilman Drive, La Jolla, CA 92093-0357, USA

## Abstract

**Background:**

We have collected a comprehensive set of multi-unit data on dissociated cortical cultures. Previous studies of the development of the electrical activity of dissociated cultures of cortical neurons each focused on limited aspects of its dynamics, and were often based on small numbers of observed cultures. We followed 58 cultures of different densities – 3000 to 50,000 neurons on areas of 30 to 75 mm^2 ^– growing on multi-electrode arrays (MEAs) during the first five weeks of their development.

**Results:**

Plating density had a profound effect on development. While the aggregate spike detection rate scaled linearly with density, as expected from the number of cells in proximity to electrodes, dense cultures started to exhibit bursting behavior earlier in development than sparser cultures. Analysis of responses to electrical stimulation suggests that axonal outgrowth likewise occurred faster in dense cultures. After two weeks, the network activity was dominated by population bursts in most cultures. In contrast to previous reports, development continued with changing burst patterns throughout the observation period. Burst patterns were extremely varied, with inter-burst intervals between 1 and 300 s, different amounts of temporal clustering of bursts, and different firing rate profiles during bursts. During certain stages of development bursts were organized into tight clusters with highly conserved internal structure.

**Conclusion:**

Dissociated cultures of cortical cells exhibited a much richer repertoire of activity patterns than previously reported. Except for the very sparsest cultures, all cultures exhibited globally synchronized bursts, but bursting patterns changed over the course of development, and varied considerably between preparations. This emphasizes the importance of using multiple preparations – not just multiple cultures from one preparation – in any study involving neuronal cultures.

These results are based on 963 half-hour-long recordings. To encourage further investigation of the rich range of behaviors exhibited by cortical cells *in vitro*, we are making the data available to other researchers, together with Matlab code to facilitate access.

## Background

Dissociated cultures of cortical cells grown on multi-electrode arrays (MEAs) have been used in many studies of network physiology because of their superior accessibility compared to *in vivo *models, in terms of electrical recording and stimulation, pharmacological manipulation and imaging. These studies described fundamental properties of network activity patterns [1-5], plasticity [6,7], learning *in vitro *[8-12], applications of cell cultures in pharmacological testing [13], and models of epilepsy [14].

Cortical cells in culture retain many of the properties found in their *in vivo *context, but important differences assuredly exist [3,8]. Therefore, the development of neuronal cultures *in vitro *deserves to be documented, as a baseline against which the results of experimental manipulations can be compared. This baseline could also be used in a future comparison with *in vivo *activity patterns. Previous investigations of development *in vitro *all agreed that population bursts are a major component of cultures' activity patterns, and have each focused on different aspects of the activity patterns exhibited by such cultures. While single-cell bursts commonly occur in dissociated cultures, the MEA literature does not focus on them, since intracellular recording techniques would be more suitable. Segev et al. [3,15] found that the statistical properties of the distribution of inter-burst intervals (IBIs) and burst sizes resembled those of avalanches. Opitz et al. [16] found that spike bursts are associated with a synchronous increase in [Ca^2+^]_i _throughout the neuronal population. Mukai et al. [17] focused on development changes in IBI values and spatial extent of bursts, and found that the complexity of burst patterns increased after about two weeks *in vitro*. Van Pelt et al. [5,18] focused on the temporal structure of the firing-rate envelope of bursts during development, finding that bursts gradually grew longer during the first weeks *in vitro*, but suddenly became very short and sharply defined after about a month. It is worth noting that population bursts have also been observed in cultures from many neural tissues, including spinal cord [19], and retina [20], as well as during mammalian development *in vivo*, in cortex [21], hippocampus [22], and thalamus [23], but here we will focus on cortical cultures.

Each of the previous papers uses different terminology to describe bursts and burst patterns, resulting in a patchwork of descriptions that is difficult to integrate. This situation is exacerbated by the wide variety of neuronal activity that neuroscientists call 'bursts'. MEA electrophysiologists mostly discuss population bursts, i.e., brief periods during which the spike rate of many cells or electrodes exceeds the baseline rate severalfold. Moreover, most of the previous studies were based on observations of small numbers of cultures from unspecified numbers of plating batches. Therefore they may have underestimated the variety of activity patterns that different cultures can exhibit. Here, we present an in-depth study of the development of burst patterns in cortical cultures over the course of the first five weeks *in vitro*, based on a dataset encompassing a total of 963 half-hour-long recordings and 36 overnight recordings from 58 cultures of five different sizes and densities. The cultures exhibited a surprisingly wide spectrum of spontaneous activity patterns, characterized by population bursts of qualitatively different shapes, sizes and interval distributions.

## Results

Dissociated neurons in culture began growing new neurites immediately after plating, and soon formed densely interconnected circuits. Starting from 3–4 days *in vitro *(div), we recorded half an hour of spontaneous activity on most days. In dense cultures (see Table 1), cells typically began firing action potentials around 4–5 div. Tonic firing persisted for the lifetime of the culture, but on top of that population bursts emerged after 4–6 div, which dominated the activity throughout the rest of development. During bursts, up to a hundredfold increase over baseline could be observed in the array-wide spike detection rate (ASDR), defined as the number of spikes detected per unit time, summed over all electrodes in the array. Increased activity during bursts was seen on all electrodes that recorded any activity at all. Moreover, direct inspection of recorded voltage traces revealed a greater variety of spike waveforms during bursts, including many apparently resulting from several partially overlapping single-unit spikes, suggesting that many more cells are active during bursts than are tonically active. Thus, it appears likely that most or all active neurons participated in bursting.

**Table 1:** Plating parameters.

	'Dense'	'Small'	'Sparse'	'Small & sparse'	'Ultra sparse'
Plating volume (*μ*L)	20	5	20	5	20
Density of suspension (cells/*μ*L)	2500	2500	625	625	156
Number of cells plated (nominal)	50,000	12,500	12,500	3,125	3,125
Culture diameter (mm)^*a*^	4.9 ± 0.4	3.1 ± 0.3	4.9 ± 0.4	3.1 ± 0.3	4.9 ± 0.4
Drop thickness (mm)^*a*,*b*^	1.69 ± 0.24	1.06 ± 0.23	1.69 ± 0.24	1.06 ± 0.23	1.69 ± 0.24
Density at 1 div (×10^3 ^cells/mm^2^)^*c*^	2.5 ± 1.5	1.6 ± 0.6	0.60 ± 0.24	0.30 ± 0.16	0.11 ± 0.06
Number of cultures followed	30	12	10	3	3
Number of batches	8	3	3	1	1

^*a*^Mean ± sample standard deviation, *N *= 3 measured drops each of 5 and 20 *μ*L.

^*b*^Measured by focusing an inverted microscope on surface of MEA and top of drop (made visible by sprinkling some dust on it), and correcting for the refractive index of the liquid.

^*c*^Mean ± sample standard deviation, based on *N *= 8, 8, 7, 3, 3 cultures. Cells in the central 0.36 mm^2 ^of each MEA were counted using digital images.

**Figure 1:**
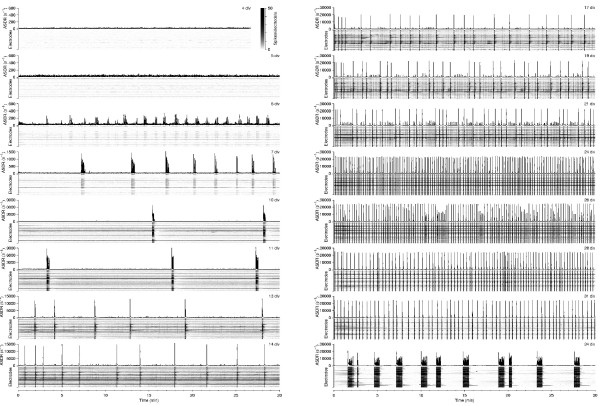
**Development of burst patterns in a dense culture**. Graphs show array-wide spike detection rates (ASDR) per second in recordings from one culture at various ages (*numbers in top right corners*). Note the different vertical scales used for each day. The spike detection rate of individual electrodes is represented by gray-scale rasters for all 59 electrodes, stacked vertically below each graph. (Each horizontal line pertains to one electrode; gray levels indicate firing rates. The same scale (see color bar next to '4 div') is used for all days. Note that the extreme increase in ASDR during bursts commonly saturates the gray scale. This representation is also used in subsequent figures.) Analogous figures from all 58 cultures used are available online.

The development of the activity of a typical dense culture is shown in Figure 1. It passed through various stages, characterized by different degrees of burstiness, different degrees of temporal clustering of bursts, different burst shapes, and different distributions of burst sizes. A particularly striking phenomenon was observed, in this case, at 7–11 div: culture-wide bursts occurred in short, sharply defined trains – or 'superbursts' – with several burst-free minutes between superbursts (DAW, Z. Nadasdy, and SMP, *in preparation*). Similar clustering was again seen at 34 div, although the internal structure of these later superbursts was different: in early superbursts, successive bursts contained fewer and fewer spikes, while in older cultures, spike counts in successive bursts within a superburst often increased (compare panels B6 and B7 in Figure 3). Note that superbursts in older cultures were a relatively rare phenomenon. Most cultures at this age exhibited more regular burst patterns; see Figure 3A.

**Figure 2:**
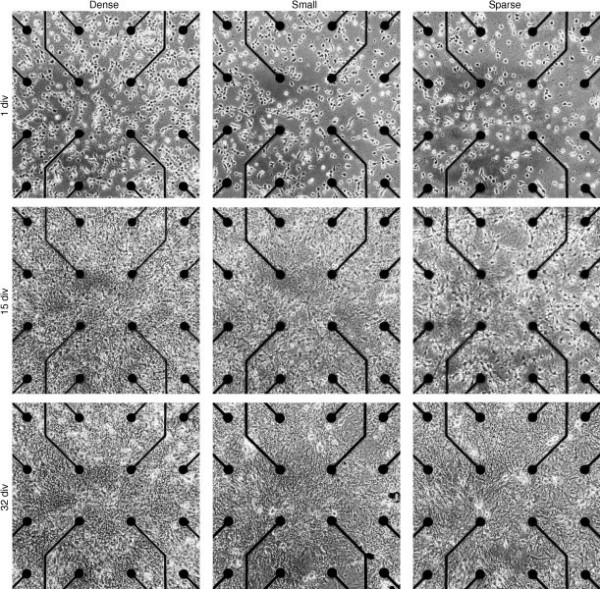
**Phase contrast micrographs of central area of cortical cultures**. Photographs are of three typical cultures of different densities, at 1, 15, and 32 days *in vitro*. Note that even our 'sparse' cultures are considerably denser than those commonly used for investigating synaptic plasticity with intracellular electrodes (see, e.g., [32]). Scale: electrode spacing is 200 *μ*m center to center.

**Figure 3:**
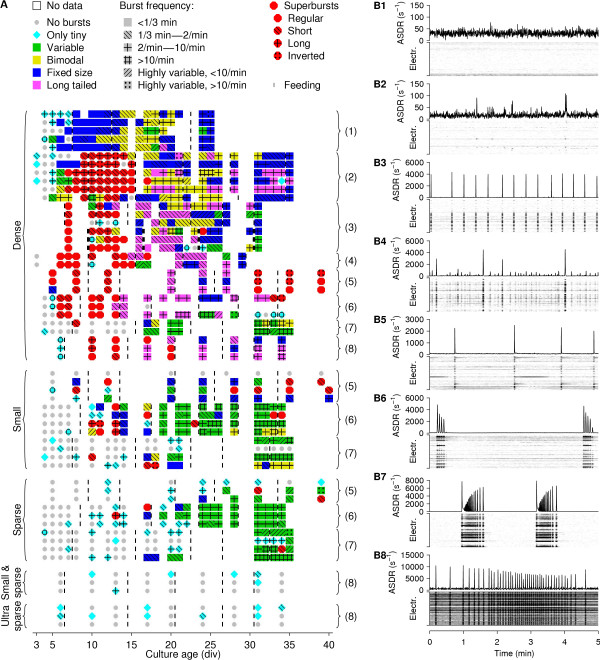
**Classification of observed bursting behaviors. A** Overview of the different classes of bursting behavior observed in our cultures. Numbers in parentheses indicate plating batch. Vertical bars indicate partial medium replacement times. Hash patterns indicate burst frequency for all types of burst patterns except superbursts. In batch 3, three cultures received full medium replacements (indicated by thicker bars in the lower three cultures of batch 3). One culture in batch 6 got infected after 20 div, and had to be discarded. **B **Examples of burst pattern classes, with array-wide spike detection rates and gray-scale rasters for all electrodes, all taken from dense cultures. **B1 **No bursting. **B2 **Tiny bursts. **B3 **Fixed size bursts. **B4 **Variably sized bursts. **B5 **Long-tailed bursts. **B6 **Regular superbursts. **B7 **Inverted superbursts. **B8 **Dramatic burst rate variation. Gray scales are as in Figure 1.

We followed cultures of five different densities (Table 1). Typical cultures of various densities are shown at 1, 15, and 32 div in the phase contrast photographs in Figure 2.

### Classification of population bursts

The quantitative details of the development of different cultures varied widely. Indeed, cultures from different platings could show qualitatively different patterns during development. For instance, superbursts were observed in only about half of all cultures. Therefore, we made analogues of Figure 1 for each of the cultures studied available online (see below, under *Additional files*). To allow easier comparison between the burst patterns exhibited by cultures of different densities and different stages of development, we devised a classification of burst patterns based on the following criteria. (For full details, see *Methods.*)

**Burstiness** Any burst spanning fewer than 5 electrodes was termed *tiny*. The first stage of classification was whether a recording (half an hour of activity) contained no bursts at all (example in Figure 3B1), only tiny bursts (Figure 3B2), or also larger bursts. Recordings with larger bursts were further classified, as follows.

**Size distribution** In some recordings, bursts had very similar sizes (Figure 3B3), in others a wide range of burst sizes occurred (Figure 3B4). Sometimes, this range was a continuum, sometimes large and smaller bursts were quite distinct, with very few bursts of intermediate size occurring. Accordingly, recordings were given the labels *fixed size, variable size*, and *bimodal size distribution.*

**Long-tailed bursts** Bursts usually had a fast onset, followed by a somewhat slower decay. Sometimes, the main part of the decay was followed by an extended period of several seconds during which several channels continued bursting, resulting in a 'tail' of elevated firing rates (Figure 3B5). If most large bursts in a recording had such tails, the label *long-tailed *was applied to it. (See *Methods *for details.)

**Burst rates** Burst patterns were further classified by the rate of occurrance of bursts. Usually, burst rates were relatively constant over time, either with regular spacing (Figure 3B3), or with more chaotic spacing (Figure 3B4). Sometimes, the burst rate varied by more than an order of magnitude over the course of a recording (Figure 3B8). In that case, the burst rate was considered *highly variable.*

**Superbursts** Many cultures went through a developmental period during which a second level of organization appeared in the temporal distribution of bursts. Bursts occurred in periodic trains of about 4–12, with several minutes of tonic firing between trains (Figure 3B6). If most large bursts occurred in such 'superbursts', the recording was labeled accordingly. Recordings with superbursts were further subclassified based on whether the number of bursts per superburst was highly conserved (*regular superbursts*), variable but typically less than ten (*short*), or variable with a higher average (*long*). Usually, the number of spikes decayed in successive bursts inside superbursts. If not, the shape was considered *inverted *(Figure 3B7). Mixtures of 'normal' and 'inverted' superbursts were never observed.

Based on this classification, a more concise overview of observed burst patterns was constructed (Figure 3A).

Array-wide synchronized bursting usually began after 5–7 div in dense cultures, and later in sparser cultures. Small bursts involving 1–5 electrodes were often observed several days before global synchronization. Burst patterns changed with culture age, and these changes were still on-going after 30 div. Thus it does not appear that cultures were truly mature at this age, in contrast to a previous report [24]. Figure 3A reveals that cultures from the same plating batch developed along strikingly parallel lines. In contrast, even between batches of the same density large differences existed both in terms of development speed and in terms of the type of burst patterns exhibited. This was most likely not due to the details of the medium replacement schedule, since the 'feeding' times (indicated in the figure by vertical thin black bars) did not coincide with marked developmental changes. Finally, the sparsest cultures we studied had very low burstiness, consistent with observations in low-density cultures used by other researchers for patch-clamp experiments in studies of synaptic plasticity.

### Development of firing rates

**Figure 4:**
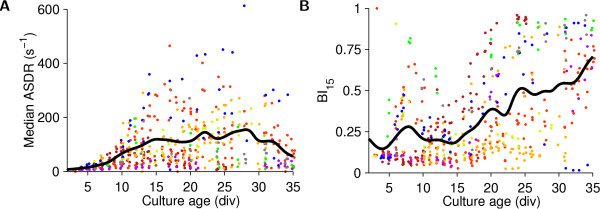
**Development of firing and bursting activity in dense cultures. A** Median ASDR as a function of developmental age. Medians were taken across all 1800 one-second-wide time bins in individual half-hour long recordings. (Since bursts occupy a small fraction of time bins, a culture's median ASDR over time is a good indication of the baseline ASDR outside of bursts.) **B **Burstiness index as a function of developmental age. Dots in **A **and **B **are measurements from individual cultures, colored by plating batch. Black lines are interpolated averages across all cultures, using a Gaussian window with a half-width of 1 day. Dots were horizontally jittered by ± 0.25 days for visual clarity.

**Figure 5:**
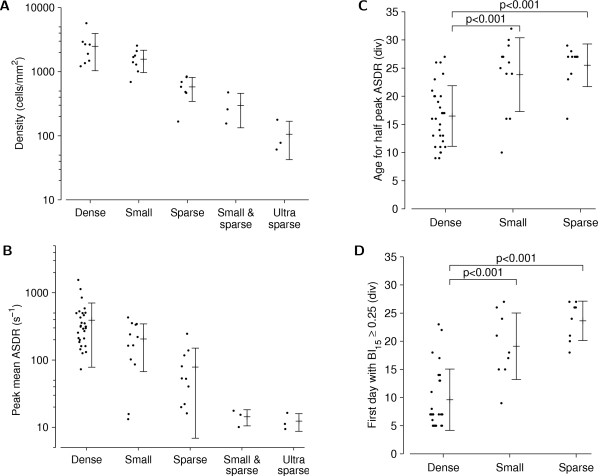
**Comparison of the development of cultures of different sizes**. **A **Actual density of cultures at 1 div. **B **Maximum (across days) of ASDR (averaged over 30 minutes of recording) observed in the first 35 div. **C **First day on which ASDR reached half of its maximum. **D **First day with burstiness index greater than 0.25. Error bars indicate the mean and the sample standard deviation. Horizontal jittering of dots is for visual clarity only. Note that the vertical scale is logarithmic in **A **and **B**, which explains why the error bars appear asymmetric. In small-and-sparse and ultra-sparse cultures, the ASDR remained so low that the age at which half of the maximum was reached could not be measured accurately, and the BI never reached 0.25. Therefore, no data are shown.

The development of network firing activity was strongly influenced by plating density. In dense cultures, the activity level (median ASDR) steadily increased during the first three weeks *in vitro*, then leveled off (Figure 4A), while the degree to which culture-wide bursts dominated the activity kept increasing (Figure 4B). In sparser cultures, not only were the ASDRs smaller than in dense cultures (Figure 5A–B), but we also found that the development of sparse cultures was delayed compared to dense cultures, both in terms of how fast their ASDR increased (Figure 5C), and even more so in terms of their burstiness (Figure 5D). A reduced ASDR in sparse cultures compared to dense cultures is to be expected, because in a sparser culture there are fewer cells in the vicinity of each electrode, and so if individual cells fire at the same rate, its ASDR would be smaller than that of a dense culture. However, the observed delay in development cannot be explained by the fact that fewer cells are in contact with electrodes, and instead indicates that cell density regulates the development of firing rates of individual cells (by a cellular or network-level mechanism).

### Development of burst duration, propagation speed, and size

**Figure 6:**
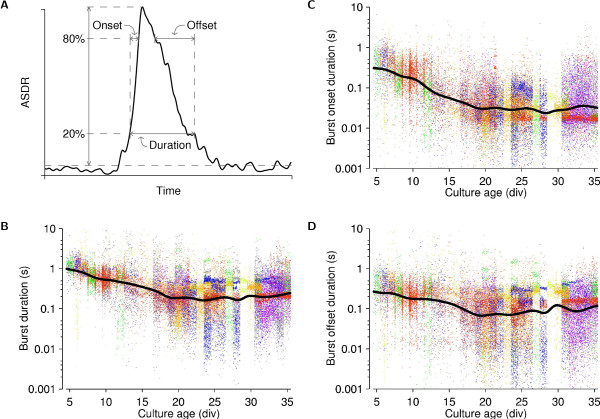
**Characterizing burst shapes in dense cultures**. **A **Parameters that define the shape of population bursts. We measured the ASDR as a function of time during the burst, smoothed with a 10 ms Gaussian filter. The 20% and 80% points between baseline and peak ASDR were determined and used to define the various phases. **B **Total duration of individual bursts. (Note log scale on y-axis.) **C **Duration of onset phases. **D **Duration of offset phases. In all panels, dots represent individual bursts, horizontally jittered for clarity, and colored by plating batch. Lines are interpolated averages, computed in log-space, using a Gaussian window with a half-width of 1 day.

We measured the time it took for bursts to spread across a dense culture (a burst's onset phase), as well as the time it takes for bursts to be extinguished (its offset phase) and the total duration of bursts over the course of development (Figure 6). In contrast to Habets et al. [25], we found marked developmental changes: The average total burst duration decreased from 1 s when bursts first appeared, to less than 200 ms after 20 div (Figure 6B). Simultaneously, the burst onset phase decreased from 300 ms to only 30 ms (Figure 6C), while the burst offset phase changed much less on average (Figure 6D). Note that substantial differences existed in both the values and variances of burst parameters of different cultures.

**Figure 7:**
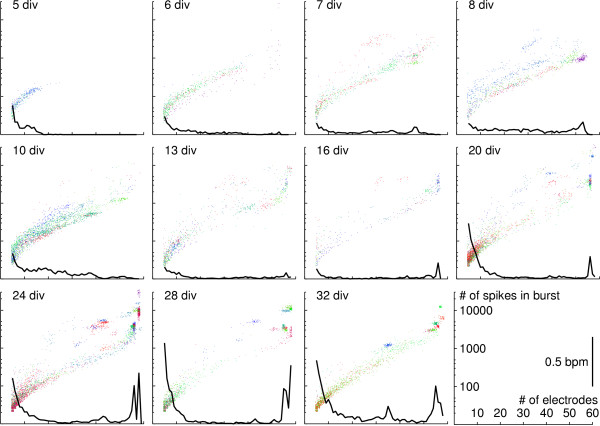
**Comparison of burst sizes during culture development**. Scatter plot of total number of spikes in burst and number of participating electrodes. Colors represent bursts from different (dense) cultures. Black traces are the frequencies (in bursts per minute; bpm) of bursts with a given number of participating electrodes, averaged across all cultures represented. Note log scale on y-axis.

The development of burst sizes, in terms of number of participating electrodes and total number of spikes, is illustrated by Figure 7. It is interesting to note how well the relationship between spike count and number of electrodes is preserved throughout most of the developmental period studied.

### Responses to electrical stimulation

**Figure 8:**
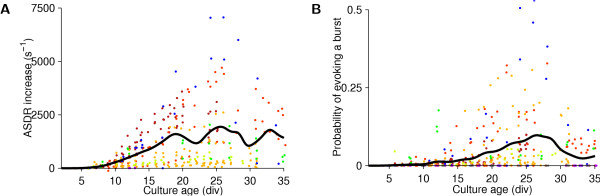
**Strength of responses to stimulation during the development of dense cultures**. **A **Increase in ASDR during the first 50 ms post-stimulus, averaged over all 50 presentations and 59 electrodes. Each dot represents a set of stimuli delivered to one culture on a given day, colored by plating batch and horizontally jittered for visual clarity. The line is the interpolated average across all cultures, using a Gaussian window with a half-width of 1 day. **B **Fraction of stimuli that evoked a burst.

Electrical stimulation has been used as a probe into network state or as a means to induce plasticity in many studies. Moreover, the combination of multisite recording and stimulation is central to our research on reembodying neuronal cultures using virtual reality or robotics [9,26]. Therefore, we investigated the connection between the development of spontaneous activity and of responsivity to electrical impulses. We found that detectable responses to stimuli appeared later in development than spontaneous activity (compare Figure 8 to Figure 4). In cultures older than two weeks, stimulation often elicited bursts, but most cultures could not be driven to burst more frequently than once every few seconds. Interestingly, the fraction of stimuli that elicited bursts decreased after about four weeks *in vitro*, even as the burstiness in spontaneous activity increased, and while the immediate response to stimulus pulses remained constant. This was not due to a greater number of spontaneous bursts during the stimulation sequence – there were none – but was due to the sizes of the bursts evoked: the total number of spikes in bursts during stimulation remained constant, even as the number of bursts decreased (data not shown).

**Figure 9:**
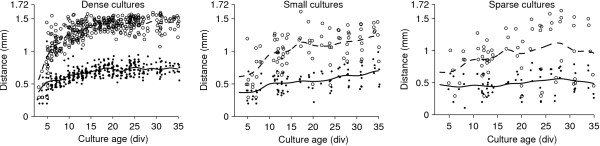
**Development of functional projections in cultures of different densities**. Median distance of sites of non-synaptic responses to stimulated electrode (*solid, dots*) and 90th percentile of distance distribution (*dashed, circle*) in individual recordings from dense culture, small cultures, and sparse cultures (*left to right*). The diameter of the MEA (maximum electrode distance) is 1.72 mm. Projections likely continued to grow beyond this length, especially in the dense cultures, but our method is incapable of following that development. (Interpolation was performed with a window half-width of 1 day for the leftmost panel, and 2 days for the other two, to obtain a smooth curve for the smaller data sets.)

Beyond the number of spikes elicited, the location of responses in relation to the site of stimulation offers information about the connectedness of the network, and the spread of functional projections. We measured the distribution of distances between stimulation sites and direct responses (see *Methods*), and found that functional projections grew rapidly during the first week *in vitro *in dense cultures, reaching across the entire array within 15 days (Figure 9). Outgrowth was slower in small and sparse cultures, and the typical length of projections after 5 weeks *in vitro *was shorter.

### Sensitivity to movement

**Figure 10:**
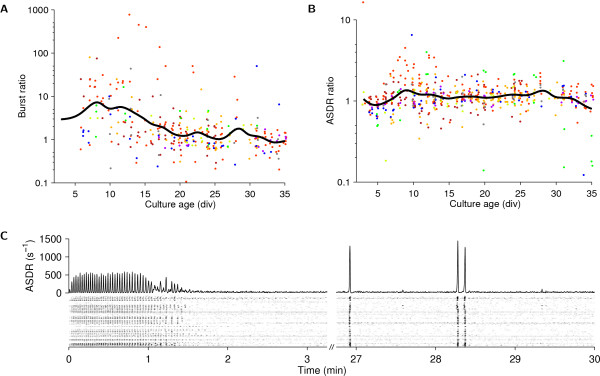
**Sensitivity of spiking activity to mild mechanical perturbation (movement of the culture dish)**. **A **Number of bursts in first minute after culture was moved into the recording device, normalized to burst rate 10–30 minutes later. **B **Mean ASDR in first minute after culture was moved into the recording device, normalized to ASDR 10–30 minutes later. **C **Example of mechanically-induced bursting, recorded at 8 div from a dense culture. Gray scale is as in Figure 1. Lines are interpolations of the data, using a Gaussian window with a half-width of 1 day.

We tested whether mild mechanical perturbation had an effect on a culture's activity, and found that indeed it did. This is important, because published results from many researchers depend on recordings made shortly after moving MEAs, since recording from multiple cultures on a day with a single recording device necessarily involves moving MEAs. Young cultures (<20 div) often responded to being moved into the recording device by firing a volley of bursts that lasted 1–2 minutes (Figure 10). Interestingly, the total rate of spikes fired in the first few minutes after moving an MEA was only slightly elevated; the mechanical perturbation increased the synchronicity between neurons almost without increasing total firing rates. We confirmed that the effect was mechanically induced – rather than by subtle differences between the recording environment and the storage shelf, 30 cm lower in the same incubator – by lifting the recording device after 15 minutes of recording and putting it back down. This resulted in another volley of bursts (data not shown). Thus, the effect was not due to environmental influences such as a possible difference in temperature. We also ruled out that exposure to light might cause the increased bursting, by moving cultures in total darkness and finding no difference between light and dark conditions (data not shown).

**Figure 11:**
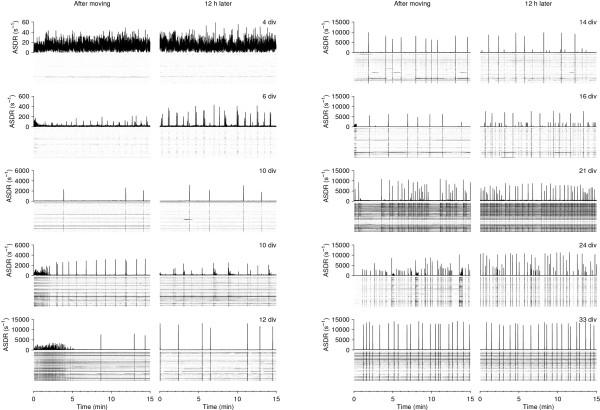
**Comparison of activity after moving a culture into the recording device with activity around 12 hours later**. Comparison of activity in first 15 minutes after moving a culture into the recording device (*left-hand graphs*) with activity in the same culture around 12 hours later (*right-hand graphs*), in cultures of various ages *in vitro*. Examples are from different cultures. Gray scales are as in Figure 1.

The observation that mechanical perturbation affects activity patterns led to the concern that perhaps recording only immediately after such a perturbation gives a distorted view of the 'typical' activity of cultures at a given age. We compared the activity patterns immediately after moving a culture with the activity about 12 hours later in our overnight recordings (*N *= 36), and found that substantial differences can indeed be observed in some young cultures (Figure 11). However, the effect was mostly limited to the first 5 minutes of recordings. Beyond that, whatever burst pattern the culture exhibited shortly after moving, this pattern was still present 12 hours later. In conclusion, our half-hour long recordings provide a view on the activity that is not significantly distorted by sampling artifact.

### Sources of variability

We observed that different cultures had some degree of maintained personality. That is, differences between cultures exceeded the day-to-day differences in the behavior of individual cultures. Moreover, even batches seem to have distinguishing features. To substantiate these observations, we quantified the sources of variability between firing rate patterns using a difference index (DI) defined as follows:

DIASDR(f1,f2)=|f1−f2|f1+f2.
 MathType@MTEF@5@5@+=feaafiart1ev1aaatCvAUfKttLearuWrP9MDH5MBPbIqV92AaeXatLxBI9gBaebbnrfifHhDYfgasaacH8akY=wiFfYdH8Gipec8Eeeu0xXdbba9frFj0=OqFfea0dXdd9vqai=hGuQ8kuc9pgc9s8qqaq=dirpe0xb9q8qiLsFr0=vr0=vr0dc8meaabaqaciaacaGaaeqabaqabeGadaaakeaacqWGebarcqWGjbqsdaWgaaWcbaGaeeyqaeKaee4uamLaeeiraqKaeeOuaifabeaakmaabmaabaGaemOzay2aaSbaaSqaaiabigdaXaqabaGccqGGSaalcqWGMbGzdaWgaaWcbaGaeGOmaidabeaaaOGaayjkaiaawMcaaiabg2da9maalaaabaWaaqWaaeaacqWGMbGzdaWgaaWcbaGaeGymaedabeaakiabgkHiTiabdAgaMnaaBaaaleaacqaIYaGmaeqaaaGccaGLhWUaayjcSdaabaGaemOzay2aaSbaaSqaaiabigdaXaqabaGccqGHRaWkcqWGMbGzdaWgaaWcbaGaeGOmaidabeaaaaGccqGGUaGlaaa@4BBA@

Here, *f*_1 _and *f*_2 _are the mean ASDRs in the two recordings. This DI is normalized to lie between 0 (if the ASDRs are the same) and 1 (if one is much larger than the other). Analogously, we computed a DI of the burstiness index from each pair of recordings. We used DIs for a number of comparisons:

**Day to day** DIs computed between all possible pairs of recordings made from the same culture on consecutive days. The mean DI at a given age is a quantitative measure of day-to-day variability at that age.

**Sister cultures** DIs computed between all possible pairs of recordings made on the same day from cultures from the same plating batch.

**Non-sister cultures** DIs computed between all possible pairs of recordings made at the same developmental age from cultures from different plating batches.

**Figure 12:**
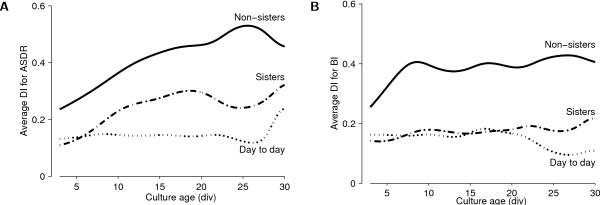
**Quantifying sources of variability in activity levels**. **A **Variability of median ASDR. **B **Variability of burstiness index. We compared the day-to-day variability for individual dense cultures with variability between sister cultures, and with variability between different platings. Interpolated using a Gaussian window with a half-width of 2 days.

This revealed that same-day differences between sister cultures were not much larger than the day-to-day differences between recordings from the same culture, and that the differences between cultures from different platings were substantially larger than differences between sister cultures (*p <*.05 at all ages except 29 and 30 div, both for mean ASDR and for burstiness index; Figure 12).

## Discussion

### The nature of bursts in culture

**Figure 13:**
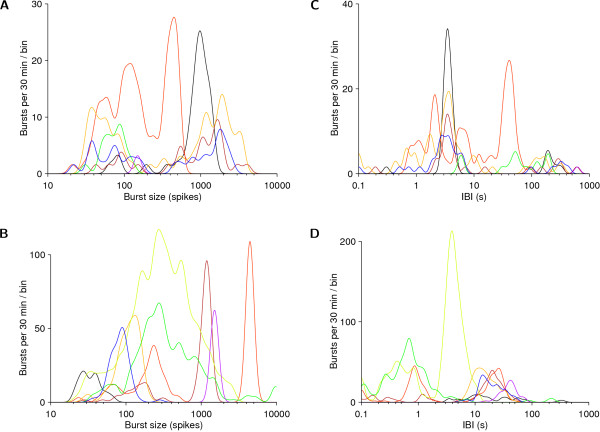
**Distribution of inter-burst intervals and burst sizes**. **A–B **Distribution of burst sizes. **C–D **Distribution of interburst intervals. Data are shown from 8 recordings from arbitrarily selected cultures (*different colors*), at around 7 div (**A **and **C**), and around 35 div (**B **and **D**). Bimodality in the IBI distribution results from temporal clustering of bursts. Data were binned using a Gaussian window in logarithmic space, bin size was 5% of a decade.

It appears that the bursts we observed depend on the existence of long-range connections in the culture, because the age at which such connections first appeared in dense cultures (5–10 div, see Figure 9) corresponded closely to the age culture-spanning bursts first appeared (see Figures 3A and 7). It has previously been reported that cortical networks in long-term slice cultures exhibit burst patterns with sizes (number of constituent spikes) governed by a Lévy distribution [4]. The dissociated cultures we followed showed very different burst patterns: in a given recording, there would be a clear distinction between tiny bursts and global bursts, the latter often having a relatively constant size (Figure 3B3 and Figure 13A–B). Furthermore, IBIs were quite narrowly distributed in most recordings (Figure 13C–D), in contrast to previous reports that they follow a scale-free distribution [15]. Moreover, burst patterns often had rich temporal structure. Examples include superbursts as well as dramatic minute-scale changes in burst rate (Figure 3B6–8). The richness of the structure of burst patterns suggests that bursts may play a role in the information processing required for development. Indeed, a prolonged suppression of bursting using tetrodotoxin (TTX) is known to perturb network formation [27].

### Importance of sampling from multiple platings

Despite our best efforts to keep conditions stable, we found substantial differences between the development of cultures from different platings. These differences may have been due to characteristics preserved from the animals from which the cultures derived, or they may have originated later during development. The fact that cross-plating variability increased with age *in vitro *(Figure 12) supports the second possibility. Whatever the root cause, the observation that cross-plating variability was larger than variability between sister cultures implies that it is crucial to use cultures from several different platings to obtain unbiased results. Therefore it is critical to report not only the number of cultures used, but also the number of platings from which these cultures stem, whenever dissociated cultures are used in network physiology experiments.

### Importance of ensuring a stable recording environment

Even though all our recordings took place inside the incubator in which the cultures were maintained, the physical act of connecting an MEA to the recording device had a substantial synchronizing effect on cultures' activity patterns (see Figure 10 and Figure 11). These effects were generally short-lived (lasting only a few minutes), but for experiments where the effect of a manipulation is compared to baseline conditions, such non-stationarity can pose serious problems. Therefore, it is important to connect MEAs to the recording device well ahead of starting experiments. Great care must be taken that the effect of pharmacological manipulations is not confounded by the effect of physical perturbations.

### Future directions

The present results generate a variety of new questions. For instance: Why does stimulation of older cultures yield fewer bursts than in younger cultures? Why does the number of spikes in a burst scale exponentially with the number of participating electrodes, rather than linearly, which one would naïvely expect? Why do sister cultures develop along more similar lines than non-sisters? Is this due to preservation of properties from the tissue from which they were obtained, or due to the inevitable slight inter-batch differences in culture maintenance? Why does mechanical perturbation transiently increase synchronization? And why does that only happen at a certain developmental stage? We have only begun to investigate these questions.

## Conclusion

By following the development of a larger number of cultures than any previous report based on MEA recordings, we have found that the range of spiking dynamics exhibited by networks of cortical cells *in vitro *is much more complex than previous publications suggest. While the activity of all dense cultures became dominated by array-wide bursts as the cultures matured, the sizes, shapes, and temporal patterns of these bursts varied widely. Indeed, the range of behaviors of these cultures is so rich that this paper can only begin to describe the diverse activity patterns present in these recordings. Therefore, we invite others to join us in the study of activity patterns of networks of cortical cells *in vitro*. To this end, we have made the entire dataset used for this paper available on the web (see *Additional files*, below). (Send access requests to: Steve Potter steve.potter@bme.gatech.edu.) Researchers may download our recordings of spike waveforms (a total of 45 GB, compressed), or reduced files containing only time stamps and electrode IDs (a total of 4 GB). Example Matlab code to efficiently access the files, and documentation are available as well.

By allowing comparison with activity patterns occurring during healthy development and disease *in vivo*, an understanding of the development of complex dynamics in the firing behavior of cortical networks *in vitro *will increase the relevance of culture models to *in vivo *neuroscience.

## Methods

### MEA preparation

We used MEAs with 59 electrodes with a diameter of 30 *μ*m, purchased from Multichannel Systems (Reutlingen, Germany). The electrodes were organized in a square grid with the corners missing, spaced 200 *μ*m center-to-center. One electrode on the edge of the array was also absent; its place was taken by a large ground electrode. MEAs were pre-treated with poly-ethylene-imine (PEI) and laminin, as previously described [8]. Laminin (0.02 mg/mL in Neurobasal medium) was applied directly to the center of the array, in drops of either 5 or 20 *μ*L. To prevent premature evaporation, drops of 15 *μ*L medium were spread around the inside edge of the culture dish, and the dishes were sealed with Teflon membranes impermeable to water [8]. Laminin drops were removed by vacuum aspiration just prior to plating cells.

### Cell culture

Cells (neurons as well as glia) were obtained from the cortices of (E18) rat embryos using procedures described before [14]. Briefly, timed-pregnant Wistar rats were sacrificed using CO_-32 _inhalation, according to NIH approved protocols, at day 18 of gestation. Embryos were removed and decapitated, and the anterior part of the cortices (including somatosensory, motor, and association areas) were dissected out. At the rostral edge, the boundary with the olfactory bulb was used as a landmark; at the caudal edge, the third ventricle and the boundary with the lateral horn of the hippocampus were used as landmarks. Striatum and hippocampus were not included. Cortices from several embryos from the same litter were combined, cut into 1mm^3 ^chunks, and dissociated using papain followed by trituration. Cells were spun down onto 5% bovine serum albumin (BSA) to remove debris, then resuspended in Neurobasal medium with 10% horse serum, and passed through a 40 *μ*m strainer. Cell density was determined using a haemocytometer. The cell suspension was stirred by pipetting with a wide-bore 1 mL tip (Hamilton, Reno, NV) before taking either 5 or 20 *μ*L and plating it on the still-wet laminin-coated area. (The cell suspension always spread out to exactly cover the wet area left by the laminin solution after it had been aspirated out.) Several plating densities were used, as summarized in Table 1. Recordings were obtained from 58 cultures from eight dissections, performed over the course of nine months.

Cultures were maintained in Teflon-sealed dishes, in an incubator with 5% CO_2_, 9% O_2_, 35°C and 65% relative humidity [8]. After 24–36 h, the plating medium was replaced by a serum-containing DMEM-based medium adapted from Jimbo et al. [7]: DMEM (Irvine Scientific) has osmolarity 330 mOsm, from 154.6mM K^+^, 1.8 mM Ca^2+^, 0.8 mM Mg^2+^; 10% Horse serum (Hyclone), 0.5 mM GlutaMax (used in preference to glutamine to avoid the toxicity associated with glutamine breakdown), 1 mM Sodium pyruvate, 0.06 IU/mL Insulin, pH: 7.3. Half of the medium was replaced approximately every five days in most experiments, as indicated by black bars in Figure 3A. To test whether feeding schedule affected activity, all medium was replaced every seven days in some experiments (*N *= 3). This did not result in significantly different activity patterns compared with sister cultures. Feeding always took place after the day's recording session, to allow at least 12 hours for transient effects to disappear before the next recording.

To improve consistency between batches, all dissociations and handling of cultures was done by a single experimenter (DAW), while dissections were performed by one lab technician (Sheri McKinney).

### Recording and stimulation

Recording took place in the same incubator used for maintaining cultures, using a pre-amplifier from Multichannel Systems (Reutlingen, Germany). Excess heat from the pre-amplifier electronics was removed using a custom Peltier-cooled platform, keeping the culture at 35°C. Recording started immediately after transferring each culture into the recording device. MEABench [28] was used for data acquisition and online spike detection. Most recording sessions lasted 30 minutes, but in some cases (*N *= 36), a recording was allowed to continue overnight, to collect a library of longer recordings, and to be able to test whether activity patterns observed shortly after moving a culture around were substantially different from activity patterns produced by the culture at other times.

After most recordings from plating batches 1–7, cultures were probed using biphasic voltage pulses of ± 0.8 V, 400 *μ*s per phase [29], applied at 0.3 s intervals sequentially to all electrodes using our custom stimulator [30]. Stimulation artifacts were removed in software using the SALPA algorithm [31]. A total of 50 pulses were delivered to each electrode per session. Whether or not cultures were exposed to electrical stimuli did not result in significantly different activity patterns compared to sister cultures (*N *= 3; data not shown).

### Data analysis

#### Spike detection

Spikes were detected online using a threshold based detector as upward or downward excursions beyond 4.5× estimated RMS noise [28]. Spike waveforms were stored, and used to remove duplicate detections of multiphasic spikes. A variety of spike waveform shapes was observed on many electrodes, but distinct clusters in waveform space were not usually seen, presumably because many cells contributed to the spike train at each electrode, especially during bursts. Also during bursts, overlapping waveforms were a common occurrence, making spike sorting problematic. Thus, sorting was not attempted, and all results in this paper are based on multiunit data. Readers with promising spike sorting algorithms are especially welcome to test them on our dataset, which is available online in its entirety.

#### Burst detection

Bursts were detected using the SIMMUX algorithm [28]. Briefly, each electrode trace was searched for *burstlets*: sequences of at least four spikes with all inter-spike intervals less than a threshold (set to 1/4 of that electrode's inverse average spike detection rate, or to 100 ms if the electrode's average spike detection rate was less than 10 Hz). Any group of burstlets across several electrodes that overlapped in time was considered a *burst.*

#### Classification of population bursts

After detection, bursts and burst patterns were classified based on the following criteria:

#### Burstiness

One or several days before culture-wide bursts first appeared, bursts on one, or sometimes two or three, electrodes were often observed. These bursts had a shorter duration and smaller spike count than culture-wide bursts, and were thus considered a separate class, termed *tiny bursts*. Tiny bursts were not further analysed. Recordings with more than just tiny bursts were further classified, as follows.

#### Size distribution

Let N* be the number of spikes in the 3rd largest burst (in a given half-hour long recording from a particular culture). Bursts with at least 34
 MathType@MTEF@5@5@+=feaafiart1ev1aaatCvAUfKttLearuWrP9MDH5MBPbIqV92AaeXatLxBI9gBaebbnrfifHhDYfgasaacH8akY=wiFfYdH8Gipec8Eeeu0xXdbba9frFj0=OqFfea0dXdd9vqai=hGuQ8kuc9pgc9s8qqaq=dirpe0xb9q8qiLsFr0=vr0=vr0dc8meaabaqaciaacaGaaeqabaqabeGadaaakeaadaWcaaqaaiabiodaZaqaaiabisda0aaaaaa@2EA6@N* spikes were termed *large*. Bursts with at least 14
 MathType@MTEF@5@5@+=feaafiart1ev1aaatCvAUfKttLearuWrP9MDH5MBPbIqV92AaeXatLxBI9gBaebbnrfifHhDYfgasaacH8akY=wiFfYdH8Gipec8Eeeu0xXdbba9frFj0=OqFfea0dXdd9vqai=hGuQ8kuc9pgc9s8qqaq=dirpe0xb9q8qiLsFr0=vr0=vr0dc8meaabaqaciaacaGaaeqabaqabeGadaaakeaadaWcaaqaaiabigdaXaqaaiabisda0aaaaaa@2EA2@N* spikes, but less than 34
 MathType@MTEF@5@5@+=feaafiart1ev1aaatCvAUfKttLearuWrP9MDH5MBPbIqV92AaeXatLxBI9gBaebbnrfifHhDYfgasaacH8akY=wiFfYdH8Gipec8Eeeu0xXdbba9frFj0=OqFfea0dXdd9vqai=hGuQ8kuc9pgc9s8qqaq=dirpe0xb9q8qiLsFr0=vr0=vr0dc8meaabaqaciaacaGaaeqabaqabeGadaaakeaadaWcaaqaaiabiodaZaqaaiabisda0aaaaaa@2EA6@N*, were termed *medium*. Non-tiny bursts with fewer than 14
 MathType@MTEF@5@5@+=feaafiart1ev1aaatCvAUfKttLearuWrP9MDH5MBPbIqV92AaeXatLxBI9gBaebbnrfifHhDYfgasaacH8akY=wiFfYdH8Gipec8Eeeu0xXdbba9frFj0=OqFfea0dXdd9vqai=hGuQ8kuc9pgc9s8qqaq=dirpe0xb9q8qiLsFr0=vr0=vr0dc8meaabaqaciaacaGaaeqabaqabeGadaaakeaadaWcaaqaaiabigdaXaqaaiabisda0aaaaaa@2EA2@N* spikes were termed *small*. If there were more medium bursts than large bursts, the burst size was considered *variable *(Figure 3B4). Otherwise, if there were more small bursts than large bursts, the burst size distribution was considered *bimodal*. If there were more large bursts than medium or small bursts, the burst size was considered *fixed *(Figure 3B3).

#### Long-tailed bursts

Non-tiny bursts with a 'tail' of at least 3 seconds during which the ASDR remained elevated by at least 50% above baseline levels were considered *long-tailed*. If at least half of all large and medium bursts in a recording were long-tailed, it was deemed dominated by long-tailed bursts (Figure 3B5).

#### Burst rates

If the highest burst rate (determined from the shortest time span containing 10 inter-burst intervals) differed from the lowest burst rate (determined from the longest time span containing only 3 inter-burst intervals) by a factor 10 or more, the burst rate was considered *highly variable *(Figure 3B8).

#### Superbursts

If at least 50% of all large and medium bursts occurred inside tight clusters (inter-cluster intervals at least 10× longer than intra-cluster intervals), the recording was deemed to be dominated by *superbursts*. If the variance of the number of bursts per superburst was small (less than half of its average), the superbursts were considered *regular *(Figure 3B6). If not, they were considered *short *if the average number of bursts per superburst was less than 10, or *long *otherwise. If the number of spikes decayed in successive bursts inside superbursts, the superburst shape was considered normal. If it grew, the shape was considered *inverted *(Figure 3B7).

These criteria were hand-picked to highlight important features of bursts, thus making the classification more intuitive than computer-selected criteria would. Note that the classification itself was performed automatically.

#### Quantification of burstiness

Burst detection is a prerequisite for quantifying burst shapes and burst patterns, but for merely describing the level of burstiness of a recording, it suffices to quantify the temporal clustering of spikes. This was done as described previously [14]: We counted the array-wide number of spikes in non-overlapping 1 s windows, and determined what fraction of the total number of spikes was contained in the 15% most active windows. Since bursts always occupied fewer than 10% of 1 s windows, this number, *f*_15_, is close to one if most spikes occur in bursts. Conversely, if spikes are evenly spaced in time, *f*_15 _is close to 0.15. We then defined our burstiness index as *BI = *(*f*_15 _– 0.15)/0.85. Thus, *BI *is normalized between 0 (no bursts) and 1 (all spikes in bursts).

#### Measuring functional projections

We previously reported that a monopolar biphasic stimulus pulse on one electrode typically evokes very precisely timed responses on a number of other electrodes that are insensitive to synapse blockers [29]. We concluded that stimulation most likely evokes action potentials in axons, which then cause recordable action potentials elsewhere along the axon, or in the cell body by antidromic transmission. By detecting these 'direct responses' and measuring their distances from the stimulation site, one can investigate the distances reached by functional projections in the culture in which the responses were recorded, up to the point where the projections reach beyond the extent of the electrode array. We determined the distribution of distances between all identified precisely timed stimulus-response pairs in a recording, and normalized it by the number of (stimulation site, recording site)-pairs existing in the array for each distance. The median distance of direct responses to stimuli applied to all electrodes of a given culture provides a measure of the typical length of projections in that culture. While biased towards short distances, because projections that extend beyond the edge of the array cannot be adequately accounted for, the 90th percentile of the distribution is a robust lower bound for the maximal length of projections in a culture.

## Authors' contributions

DAW collected all data, performed the analysis, and prepared text and figures for the manuscript. JP contributed to the design of the study and to the preparation of the manuscript. SMP was instrumental to the conception of this study, helped interpret the results, and contributed to the preparation of the manuscript.
